# Dissolvable Microneedle Patches to Enable Increased Access to Vaccines against SARS-CoV-2 and Future Pandemic Outbreaks

**DOI:** 10.3390/vaccines9040320

**Published:** 2021-04-01

**Authors:** Jesse O’Shea, Mark R. Prausnitz, Nadine Rouphael

**Affiliations:** 1 Hope Clinic of the Emory Vaccine Center, Division of Infectious Diseases, Department of Medicine, School of Medicine, Emory University, 500 Irvin Court, Suite 200, Decatur, Atlanta, GA 30030, USA; nroupha@emory.edu; 2 School of Chemical & Biomolecular Engineering, Georgia Institute of Technology, Atlanta, GA 30332, USA; prausnitz@gatech.edu

**Keywords:** dissolvable microneedle patch, drug delivery, skin vaccination, vaccine delivery, equity

## Abstract

Vaccines are an essential component of pandemic preparedness but can be limited due to challenges in production and logistical implementation. While vaccine candidates were rapidly developed against severe acute respiratory syndrome coronavirus 2 (SARS-COV-2), immunization campaigns remain an obstacle to achieving herd immunity. Dissolvable microneedle patches are advantageous for many possible reasons: improved immunogenicity; dose-sparing effects; expected low manufacturing cost; elimination of sharps; reduction of vaccine wastage; no need for reconstitution; simplified supply chain, with reduction of cold chain supply through increased thermostability; ease of use, reducing the need for healthcare providers; and greater acceptability compared to traditional hypodermic injections. When applied to coronavirus disease 2019 (COVID-19) and future pandemic outbreaks, microneedle patches have great potential to improve vaccination globally and save many lives.

## 1. Introduction

Many of the great pandemics in the 20th and 21st centuries have been caused either by an influenza or coronavirus. Many experts have warned that repeated pandemics are inevitable and likely imminent [1]. Vaccines are an essential component of pandemic preparedness but can be limited due to challenges in production and logistical implementation. Now, in one year, the coronavirus disease 2019 (COVID-19) pandemic, caused by severe acute respiratory syndrome coronavirus 2 (SARS-CoV-2), has led to 100 million confirmed infections and more than 2 million deaths worldwide [2].

The COVID-19 pandemic has overwhelmed both health and economic systems. While vaccine candidates were rapidly developed against SARS-COV-2, immunization campaigns remain an obstacle to achieving herd immunity. In previous pandemics, some countries have had trouble accessing vaccines and other essential health products [3]. Overwhelming demand, scarce manufacturing capacity, high costs, dependency on cold chain supply, and lack of global allocation mechanisms have played a role in those delays [3]. Most vaccines for SARS-CoV-2 are injected using a hypodermic needle and require multiple doses. For example, authorized vaccines including mRNA-based technology, such as Moderna’s mRNA-1273, Pfizer/BioNTech’s Comirnaty, and other vaccines like Oxford/AstraZeneca’s AZD1222, Johnson & Johnson’s Ad26.COV2.S, Sinovac’s CoronaVac, Sinopharm’s BBIBP-CorV, CanSinoBIO’s Convidecia, Gamaleya’s Sputnik V, BEKTOP’s EpiVacCorona, and Bharat Biotech’s Covaxin, mostly require two doses administered by specially trained healthcare providers and have cold chain distribution requirements ranging from −70 °C to 8 °C, which presents significant logistical limitations [4,5,6,7].

Given these challenges, in addition to regional protectionism, the implementation of vaccination programs could be jeopardized. The founding of the COVID-19 Vaccines Global Access (COVAX) Facility by Gavi, the Coalition for Epidemic Preparedness Innovations (CEPI), and the World Health Organization (WHO) is an attempt to garner resources and unite higher- and lower-income countries for a coordinated, rapid, transparent, and equitable access to COVID-19 vaccines worldwide.

The WHO identified vaccine hesitancy as one of the top ten global health threats in 2019 [8]. Vaccine hesitancy is further magnified by needle-phobia, pain, fear of complications, and fear of leaving quarantine to a healthcare setting [9]. National surveys during the COVID-19 pandemic report that roughly 30% of adults are not sure whether they would be vaccinated and 10% did not intend to be vaccinated [9,10]. The spread of bloodborne pathogens by needle re-use is also a major concern, along with a shortage of healthcare providers, especially in developing countries. New and innovative vaccine technology and delivery mechanisms may assist in addressing these challenges.

**Figure 1:**
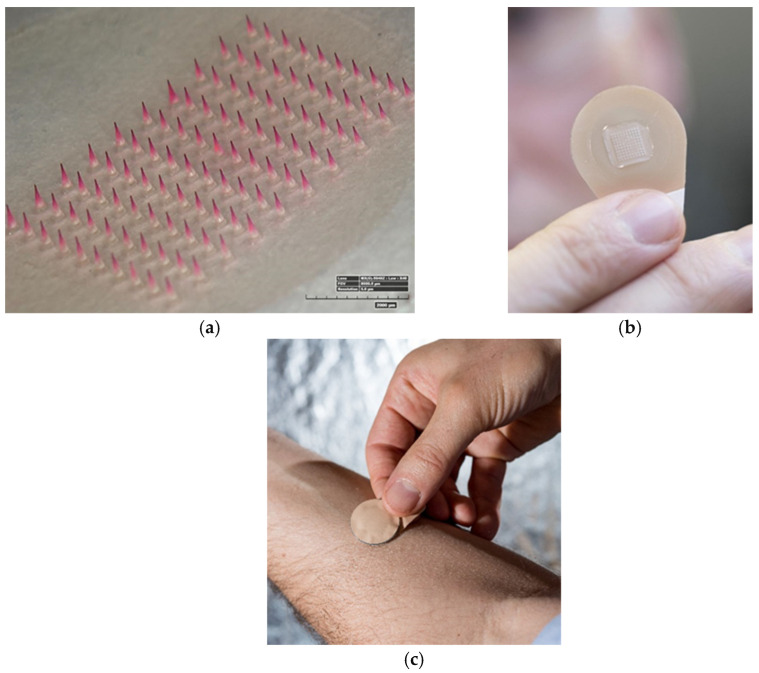
Dissolvable microneedle patch for simplified vaccination. (**a**) An array of microneedles containing pink dye to simulate vaccine. A microneedle patch (**b**) showing the microneedle array, adhesive backing, and non-adhesive tab for handling. (**c**) A microneedle patch being applied to the skin. Images courtesy of (**a**) Devin McAllister, Georgia Tech, (**b**) Christopher Moore, Georgia Tech, and (**c**) Rob Felt, Georgia Tech.

As a possible solution for pandemic countermeasures, dissolvable microneedle patches should be considered as a vaccine delivery method (Figure 1). The patches consist of micron-scale solid conical structures made of dissolvable excipients on a skin patch backing that deliver vaccine antigens across the stratum corneum barrier into the viable epidermis and dermis of the skin. As microneedles are less than one millimeter long, they cause little or no pain and are strongly preferred over traditional immunization by injection [11]. Further, microneedle patches require no special training to be administered, do not generate biohazardous sharps waste, and can be formulated for thermostability [11].

This paper examines the potential ways that microneedle patches may overcome limitations in traditional vaccination campaigns by hypodermic injection and review their applications to the current COVID-19 pandemic and beyond.

## 2. Discussion

### 2.1. Overcoming Barriers to Effective Vaccination

There are several barriers for effective vaccination campaigns in all resource settings, including the need to increase vaccine immune response, simplify the supply chain, eliminate biohazardous waste, improve cost-effectiveness, and reduce reliance on trained healthcare providers [12].

Microneedle patches have been studied for their potential application against many pathogens, including other respiratory viruses (see Table 1). For seasonal influenza, vaccination by microneedle patches resulted in faster virus clearance in the lungs of murine models [13]. For pandemic influenza, higher immunogenicity was noted in animal models compared to intramuscular injection [14]. Delivering vaccines in the epidermis or dermis puts the antigen in close contact with the skin’s rich population of antigen-presenting cells and can result in lower doses of antigens being used. Vaccinations using microneedle patches have demonstrated dose-sparing in clinical studies [15]. The use of microneedle devices ensures a more accurate, effective, and reproducible delivery of vaccine to the skin compared to injections [11]. Our clinic recently conducted a first-in-human clinical trial where outcomes after inactivated influenza vaccination by intramuscular injection were compared to dissolvable microneedle patch, which revealed similar antibody response robustness and better acceptance [16,17].

A large healthcare workforce is required to have mass vaccination campaigns, which can be limited in developing countries and could cause crowding in the pandemic era. Most vaccines that are administered by hypodermic needle and syringe injection require a trained healthcare provider to administer the vaccine. Microneedle patch vaccination allows for administration by minimally trained personnel, including self-administration, which could dramatically hasten roll-out and dissemination as well as reduce the burden on the healthcare system. Acceptability studies using pressure sensitive microneedle patches with an auditory force feedback indicator found that participants reported little to no pain with self-administration and overwhelmingly preferred microneedle patches over intramuscular injections [76].

Microneedle patches reduce the risk of sharps and sharps waste because the microneedles disappear after dissolving in the skin. The risks with sharps include unintentional re-use, needlestick injuries, and cross contamination. Up to 3.62 per 100,000 vaccinations result in needle-stick injury. The spread of bloodborne pathogens is a major concern, with an estimated 1.3 million deaths resulting from needle re-use according to WHO estimates, especially in developing countries [77,78]. Further, dissolvable microneedle patches are a safe delivery method, with no reports to date of accidental infection in controlled studies and widespread use in commercial cosmetic products [79,80,81].

Standard hypodermic needle vaccination may be wasteful via multi-dose vials and the need for reconstitution. In general, vaccine wastage rates increase as the number of vaccine doses per vial increases. Estimates suggest wastage rates for 10-dose vials may be as high as 25% for liquid vaccines and 40% for lyophilized vaccines [15,82]. Single-use microneedle patches remove this waste seen in multi-dose vials. Some vaccines need vaccine reconstitution with a diluent, which not only requires a trained healthcare provider to perform but also adds more needles, syringes, and vials that need to be safely stored and transported [15]. Microneedle patches do not require reconstitution.

Microneedle patches have improved stability and can often be stored at ambient temperature, eliminating the cold chain, and allowing for easier stockpile and storage [15,82]. Further, the patches are much smaller in size than vaccine vial and needle-syringe systems, facilitating storage and distribution, and thereby simplifying the supply chain.

The cost of vaccination is the cost of vaccine plus the logistical costs associated with making the vaccine available for use. Healthcare providers, waste disposal, vaccine storage, transportation, cold chain, and vaccine wastage all contribute to the cost of vaccination. While vaccine manufacturers often sell vaccines at significantly reduced cost for use in developing countries, the logistical costs to vaccinate can remain a significant barrier. Analyses suggest that the use of self-administered microneedle patches could not only improve vaccination coverage but would also be cost-effective [83,84]. The cost of microneedle patch manufacturing is expected to be lower than pre-filled syringes because the materials are generally low-cost medical-grade polymers and other excipients used in very small amounts. A representative microneedle array weighs less than 1 g, and the backing, adhesive, and packaging are usually made of conventional pharmaceutical supplies used in transdermal patches and other medical products [15].

Limitations of dissolving microneedle patch delivery systems for vaccines exist, including theoretical issues with dosage accuracy; inability to deliver large doses of medications (which could be an issue if using certain adjuvants require milligram doses); possibility of skin irritation and external environment affecting delivery, such as hydration of the skin or excessive sweating; and uncertainty about cost and capability of large-scale manufacturing [13]. These limitations have not presented significant issues in human clinical trials or other studies to date.

### 2.2. COVID-19 Microneedle Applications

The science of microneedle patches is robust, as shown by the many different licensed and experimental vaccines delivered (see Table 1). Four weeks after the identification of the SARS-CoV-2 S1 sequence, Kim et al. designed carboxymethyl cellulose-based dissolvable microneedle patches containing Middle East Respiratory Syndrome Coronavirus subunit MERS-CoV-S1 and SARS-CoV-2 vaccines capable of generating potent antigen-specific IgG responses [68]. The MERS-CoV-S1 vaccines induced stronger humoral responses than traditional needle injections and resulted in stronger IgG responses than via subcutaneous injection [68]. For MERS-CoV-S1, which started prior to the SARS-CoV-2 portion of the study, antibody levels continued to increase over time in mice vaccinated by microneedle patch—up to when the experiment ended at 55 weeks [68]. In another study, Kuwentrai et al. successfully designed and used dissolving microneedle patches based on a mixture of the receptor-binding domain (RBD) spike proteins and low-molecular weight hyaluronic acid (HA) together with an aluminum hydroxide gel adjuvant using a micro-molding method [34]. The team found specific B-cell antibodies and IFN-γ T-cell responses for up to 97 days after administration, though with high variation of antibody titers compared to subcutaneous injection [34]. The potential advantages of dissolvable microneedle patches for COVID-19 can be found in Table 2.

Recently, the US government through the Biomedical Advanced Research and Development Authority (BARDA) funded a total of $1.9 million to three groups developing microneedle skin patches [85]. The patches will contain the SARS-CoV-2 spike protein—the basis of nearly all COVID-19 vaccines. The patches are intended to be shelf-stable, self-administered, and self-boosting by releasing the spike protein into the body as pulses or continuously over a few weeks. This approach could eliminate the need for repeated vaccinations. Several universities and companies have announced that they are initiating pre-clinical studies for a SARS-CoV-2 vaccination using microneedle patches, but no data are available yet.

In response to the COVID-19 outbreak, there was an unprecedented effort to develop new vaccines with remarkable speed. The current bottleneck, however, is the rapid distribution and administration of the vaccines to achieve herd immunity in the global population. This depends not only on manufacturing sufficient vaccine supply, but also on the logistical and fiscal challenges of global distribution, sometimes complex cold chain requirements, and the need for skilled human capital.

Microneedle patch vaccination can ease these limitations of traditional vaccination methods, especially in resource-limited settings, although the slow pace of vaccination even in advanced economies points to the need for simplified vaccination mechanisms worldwide. However, microneedle patches for SARS-CoV-2 vaccination are currently not available due in large part to a lack of existing manufacturing and regulatory infrastructure needed for rapid development. Continued and expanded investment in innovative vaccine delivery platforms such as microneedle patches is needed to ensure the technology and infrastructure are in place for the pandemic needs of the future.

## 3. Conclusions

Microneedle patch immunization has the potential to overcome many factors affecting the uptake and distribution of traditional hypodermic intramuscular injection campaigns. Dissolvable microneedle patches are advantageous for many possible reasons: improved immunogenicity; dose-sparing effects; expected low manufacturing cost; elimination of sharps; reduction of vaccine wastage; no need for reconstitution; simplified supply chain, with reduction of cold chain supply through increased thermostability; ease of use, reducing the need for healthcare providers; and greater acceptability compared to traditional hypodermic injections. When applied to COVID-19, microneedle patches have great potential to improve vaccination globally and save many lives. While the timeline for COVID-19 microneedle patch vaccine deployment may be a missed opportunity for the current pandemic, there is a need for investment today to be better prepared for tomorrow’s pandemic needs.

## Figures and Tables

**Table 1:** Vaccines studied using dissolvable microneedle patches.

Nucleic Acid	Protein-Based (Including Virus-Like Particle (VLP))	Inactivated/Live Attenuated	Viral Vector
Ebola [18]	Diphtheria [19,20,21]	Adenovirus [22,23,24]	HIV [25,26,27]
Hepatitis B virus [28]	EV71 hand-foot-and-mouth disease (HFMD) [29]	Influenza [16,30,31,32,33]	Middle East Respiratory Syndrome (MERS-CoV-S1) [34]
Porcine circovirus type 2 [35]	Hepatitis B [36,37,38]	Measles [39,40]	Zika [41]
Rabies [42]	Human immunodeficiency virus (HIV) [43]	Modified vaccinia virus Ankara (MVA) [22]	
Tuberculosis bacillus Calmette–Guérin (BCG) [44]	Human papillomavirus infection (HPV) [45,46]	*Neisseria gonorrhoeae* [47]	
	Herpes simplex virus 2 [48]	Poliovirus [49,50,51]	
	Influenza [19,22,52,53,54,55,56,57,58,59,60,61,62,63,64]	Pseudomonas aeruginosa [65]	
	Leishmania [66]	Rotavirus [67]	
	Malaria [19]	Rubella [39]	
	SARS-2-CoV [34,68]	Streptococcus [69]	
	Staphylococcal [70]	Tuberculosis bacillus Calmette–Guérin (BCG) [71]	
	Scrub typhus [72]		
	Tetanus (toxoid) [20,73,74]		
	*Mycobacterium tuberculosis* [75]		
	Zika [41]		

**Table 2:** Potential advantages of dissolvable microneedle patch vaccine for coronavirus disease 2019 (COVID-19).

Increased Immunogenicity
Faster virus clearance
Dose-sparing effect
Reduction in vaccination wastage
Avoidance of reconstitution
Increased acceptance and less hesitancy
Little or no pain
Self-administration and reduced need for healthcare workforce
Reduced risk of sharps injury and contamination
Improved stability
Less reliance on cold chain
